# Chemical Exposures: Will DEA Findings Wash?

**DOI:** 10.1289/ehp.114-a636

**Published:** 2006-11

**Authors:** Cynthia Washam

A common soap and shampoo ingredient restricted in Europe for its suspected link to cancer is raising new concerns as study results suggest it can thwart brain development in mice. Researchers at the University of North Carolina at Chapel Hill reported in the August 2006 *FASEB Journal* that diethanolamine (DEA) irreversibly damaged the memory capacity of animals exposed before birth.

Author Steven Zeisel believes DEA could induce fetal neural abnormalities in humans, too. “It’s hard to estimate human exposure, but we believe the mice had exposures about ten times higher,” he says, assuming that people bathe and shampoo daily with DEA-containing products, and use DEA-containing sunscreen. “There’s no reason to believe we wouldn’t see similar effects in humans.” The authors note, however, that dermal absorption of DEA is less efficient in humans than in rats. Further, most DEA used in personal care products is conjugated with fatty acids, which may not have the same effects as just DEA.

Procter & Gamble principal scientist Tim Long calls Zeisel’s estimates of human exposure “grossly inaccurate,” saying that exposures from consumer product uses are actually thousands of times lower. John Bailey, executive vice president for science at the Cosmetic, Toiletry, and Fragrance Association, points out that “when you look at the exposure [of] humans, [you must take] into account the ability of the skin to protect against exposure and the fact that shampoos are rinsed off.”

DEA and its condensates are used as foaming agents in many personal products. According to the public education group known as the Cancer Prevention Coalition, DEA by itself is not harmful, but it can combine with other ingredients in cosmetics to form *N*-nitrosodiethanolamine, which the National Toxicology Program (NTP) has deemed reasonably anticipated to be a human carcinogen. According to the International Agency for Research on Cancer, levels of *N*-nitrosodiethanolamine in personal care products have declined substantially since the 1980s.

DEA first drew attention a decade ago, when animal studies suggested it might be carcinogenic. Both the International Agency for Research on Cancer and the NTP have considered listing it as a carcinogen, but ultimately decided there was too little evidence of human carcinogenicity. The more cautious European Union opted to limit the concentration of DEA allowed in personal care products sold there to 1%. In his article, Zeisel cited a 2002 NTP report stating that products sold in the United States may contain up to 25% DEA, although Bailey claims U.S. products contain 1% or less.

Zeisel and his colleagues exposed fetal mice by painting DEA dissolved in ethanol on a shaven patch of their mothers’ skin for 11 days. DEA inhibited cell development and increased cell apoptosis in the hippocampus of the fetal mice. Zeisel says such abnormalities would permanently impair the mice’s memory.

Zeisel acknowledges that even if effects are similar in humans, most babies exposed to DEA before birth would probably escape ill effects, though others may be more vulnerable to harm.

He bases this belief on his 10-year studies of the nutrient choline. The body uses choline to produce acetylcholine. Zeisel and his colleagues found that adequate choline is crucial for fetal brain development. They also discovered that individuals’ choline needs vary. In a study published in the July 2006 *FASEB Journal*, the investigators found that some human subjects placed on a choline-deficient diet quickly suffered liver and muscle dysfunction, while others did not. They traced the effects to a genetic polymorphism that raises people’s choline requirements. Zeisel says most men and about half of women carry this inherited trait. Because the DEA molecule is similar to choline, Zeisel speculated it could perturb choline metabolism and cause the same effects as choline deficiency.

“We’re not saying mothers shouldn’t shampoo their hair or use sunscreen during pregnancy,” he says. “Just look at the label. Plenty of products don’t use DEA.”

## Figures and Tables

**Figure:**
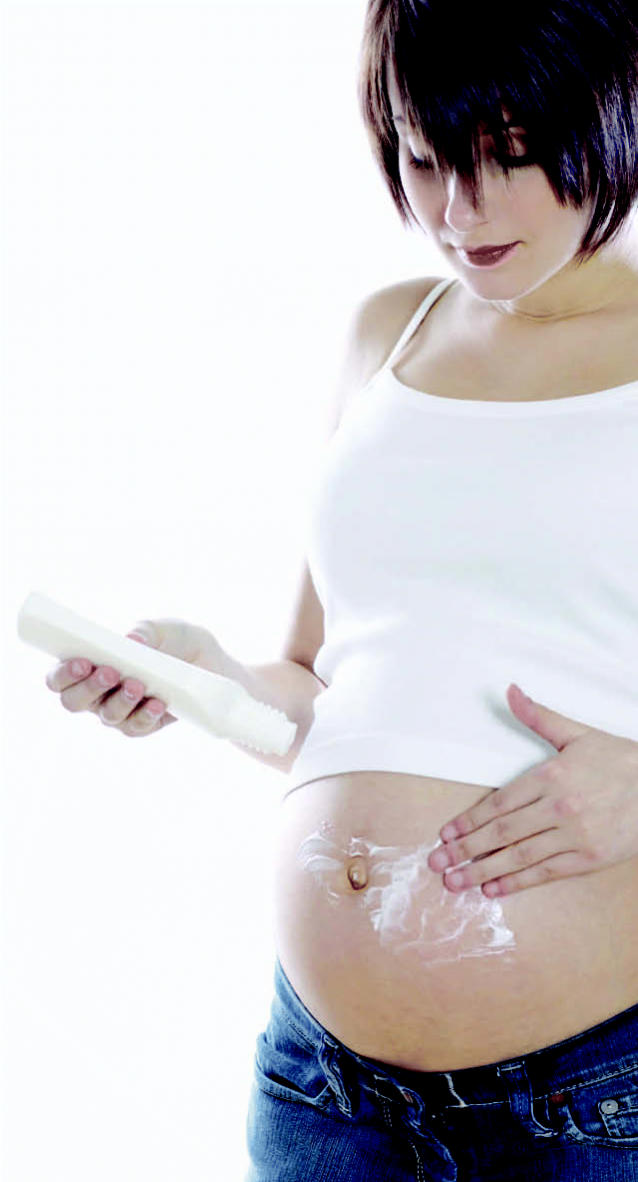
Lotion notion New data suggest more study is needed on dermal absorption of DEA by pregnant women.

